# Controlling stimulus ambiguity reduces spurious creative ideation variance in a cyclic adaptation of the alternative uses task

**DOI:** 10.1038/s41598-024-63225-2

**Published:** 2024-05-31

**Authors:** Olga Witczak, Iga Krzysik, Katarzyna Bromberek-Dyzman, Guillaume Thierry, Rafał Jończyk

**Affiliations:** 1 Adam Mickiewicz University, Poznań, Poland; 2 Bangor University, Bangor, UK

**Keywords:** Psychology, Human behaviour

## Abstract

In the alternative uses task (AUT), a well-established creativity assessment, participants propose alternative uses for common items (e.g., a brick) within a 2–3 min timeframe. While idea evaluation is likely involved, the emphasis is strongly on idea generation. Here, we test the value of presenting a word overlapping an image compared to a word only prompt, and we introduce a cyclic adaptation of the AUT explicitly calling on participants to choose their best idea. In Experiment 1, as compared to word only, word + image prompts increased idea fluency but reduced idea originality and variability within a group of native Polish speakers. Thus, word + image prompts improve AUT baselining. In Experiment 2, different participants produced as many ideas as possible within two minutes (List) or their single best idea at the end of each of three 30 s ideation cycles (Cycle). Although originality did not differ between List and Cycle overall, the first three ideas in List were rated as less creative than the ideas in Cycle. Overall, we conclude that using disambiguating images reduces spurious interindividual variability in the AUT while introducing idea evaluation in the task allows us to assess creativity beyond idea generation.

## Introduction

Creativity is commonly defined as a distinctively human capacity to produce novel and context-appropriate outputs in response to prompts or problems^1,2^. Given that creativity is a complex cognitive skill, dissecting and systematically evaluating it presents a considerable challenge^3^. Establishing a singular definition of creativity is also difficult because of its unseen, nonverbal, and unconscious characteristics. Consequently, creativity was once deemed one of “the vaguest, most ambiguous, and most confused terms in psychology”^4^.

The concept of creativity hinges on two prerequisites of originality and effectiveness; originality implies that generated ideas are unique and uncommon, and effectiveness means that they have to be appropriate and plausible in a given context^5,6^. An unusual idea with no real-life application is useless; conversely, a conventional albeit practical solution is not creative, by definition. The hallmarks of creativity can be further extended to encompass such qualities as an element of surprise, novelty, and authenticity, as well as utility and aesthetics^7,8^.

Although creativity is usually evaluated based on outputs, it must be kept in mind that the creative process is dynamic and elusive due to constant alternation between idea generation and evaluation^9^. Two key sub-processes involved are thus divergent and convergent thinking. Divergent thinking refers to the ability to generate novel ideas and consider answers to open-ended questions without much evaluation, while convergent thinking refers to evaluation and selection mechanisms aiming to identify the most suitable, effective, and practical solution(s) from an array of alternatives^10–12^. Often, these two modes of thinking have been conceptualised as lying at opposite ends of a continuum, even though they can coexist and work synergistically to enhance creative thinking^3,10, 13^.

Measures conventionally used to assess divergent thinking require participants to produce a number of ideas in response to a verbal or visual prompt. The obtained production is then assessed for fluency (i.e., number of ideas), and originality, focusing on uniqueness^14^. Measures of convergent thinking test the ability to perceive and acknowledge similarities between words, geometrical figures, or pictures, and responses are generally scored based on accuracy and ability to solve a problem within a given time limit^15–17^. One of the most established measures of creativity and, more specifically, divergent thinking, is the alternative uses task (AUT)^11^. In the AUT, participants are asked to generate as many unconventional uses for a familiar item as possible (usually presented as a word^6,18–22^), within a time limit (usually two minutes). Responses can then be evaluated for originality, flexibility, fluency, and elaboration by a pool of independent raters^18^.

One of the most robust phenomena observed in AUT studies is the serial order effect, that is, an increase in the creativity of generated ideas over time. This phenomenon could be linked to spreading of activation in semantic and episodic memory (but see Vartanian et al.,^23^). First, close and well-entrenched memory associations are retrieved, then activation spreads to more distant, weakly related concepts, leading to more unconventional alternative uses^24^. Gilhooly et al.^21^ further suggested that responses based on memory (pre-known, conventional, stored) tend to be given before responses associated with novelty (e.g., object properties, broad use, and disassembly). Moreover, they noted that memory responses are associated with lower cognitive load compared to other types of answers.

It is surprising, however, that most studies using the AUT have relied on (a small number of) words as prompts to refer to objects that can substantially vary in terms of shape, texture, colour, or size, and that can have different physical properties in terms of weight, breakability, elasticity, etc. depending on the materials from which they are made. Such under-specification likely results in spurious variability in the output for different participants, because default expectations about the nature and properties of objects introduced by the word prompt may vary substantially between individuals, contexts, and cultures. One way to mitigate this problem is to use a picture as prompt instead of a word. However, images can yield ambiguity of their own relating to low naming agreement and object prototypicality, as well as other kinds of under-specification relating to physical appearance.

Several studies have shown that images as prompts affect ideation and creativity in design sketching, word association, or alternative uses tasks^25–27^. Chrysikou et al.^28^, for instance, presented participants with either the name of an everyday object, an image depicting the object, or a combination of both (image overlaid with a word, henceforth word + image). Participants were asked to generate common uses, common alternative uses, or uncommon alternative uses. Images elicited more normative responses than words, with word + image prompts falling in between. Recently, George et al.^29^ asked participants to predict their expected creative performance in the AUT when presented with either word + image or word prompts. Participants anticipated that they would be more creative in the word + image condition, but the group of participants who actually performed the AUT on the same items generated ideas that were less original in the word + image than the word only condition. However, it remains unclear whether it is originality that suffers when using word + image prompts or whether participants appear less variable in their output due to a more uniform starting point, that is a shared understanding of what the word prompt refers to.

Another dimension of measurement associated with the AUT is fluency, that is, the number of ideas generated within a given time window. Benedek and Neubauer^30^ proposed that the more people are creative, the faster they are at providing responses thanks to greater associative fluency. Some studies have criticised fluency for its shallow explanatory potential. Beaty and Johnson^31^, for instance, referred to fluency as “a proxy of generative ability” and criticised its lack of reliability due to low inter-item correlations in AUT^32^. Such low correlation could be attributed to variability of object characteristics^33^ as well as word frequency^34^. We contend that such issues are less likely to arise and that correlations should be higher when words are presented together with a disambiguating image (see Experiment 1). Furthermore, the reason why fluency measures have poor reliability in the AUT may relate to the lack of consideration given to idea evaluation (see Experiment 2). If participants are unconstrained regarding idea quantity, then the value of fluency as a creativity measure is naturally diminished.

In the current study, we thus aimed to resolve two limitations of the classic AUT: (i) the ambiguity induced by a word only prompt; (ii) the lack of focus on idea evaluation. In Experiment 1, we compared the creative output in the form of lists of ideas from participants tested using word only and word + image prompts. Participants were randomly assigned to one of three experimental procedures: uniform with word only prompts, uniform with word + image prompts, or dual, consisting of half word only prompts and half word + image prompts (to allow for within-subject comparisons). In Experiment 2, we compared the classic list-based AUT with a new cycle procedure in which participants had to report their *single best* idea after each of three 30 s ideation cycles. Participants were randomly assigned to one of three experimental procedures: uniform with list trials (classic AUT task over two minutes), uniform with cycle trials (three cycles per item), or dual (consisting of half list and half cycle trials, allowing for within-subject comparisons). We hypothesised that:

1. Word + image prompts reduce inter-individual variability and thus provide a better baseline than word only prompts;
2. Word + image prompts tend to constrain creativity and thus reduce originality and/or fluency as compared to word only prompts;
3. Originality is higher for ideas generated in the cycle procedure than the list procedure;
4. Originality increases gradually with each consecutive cycle in the cycle procedure.

## Results

### Experiment 1: AUT items as word only or word + image

**Figure 1:**
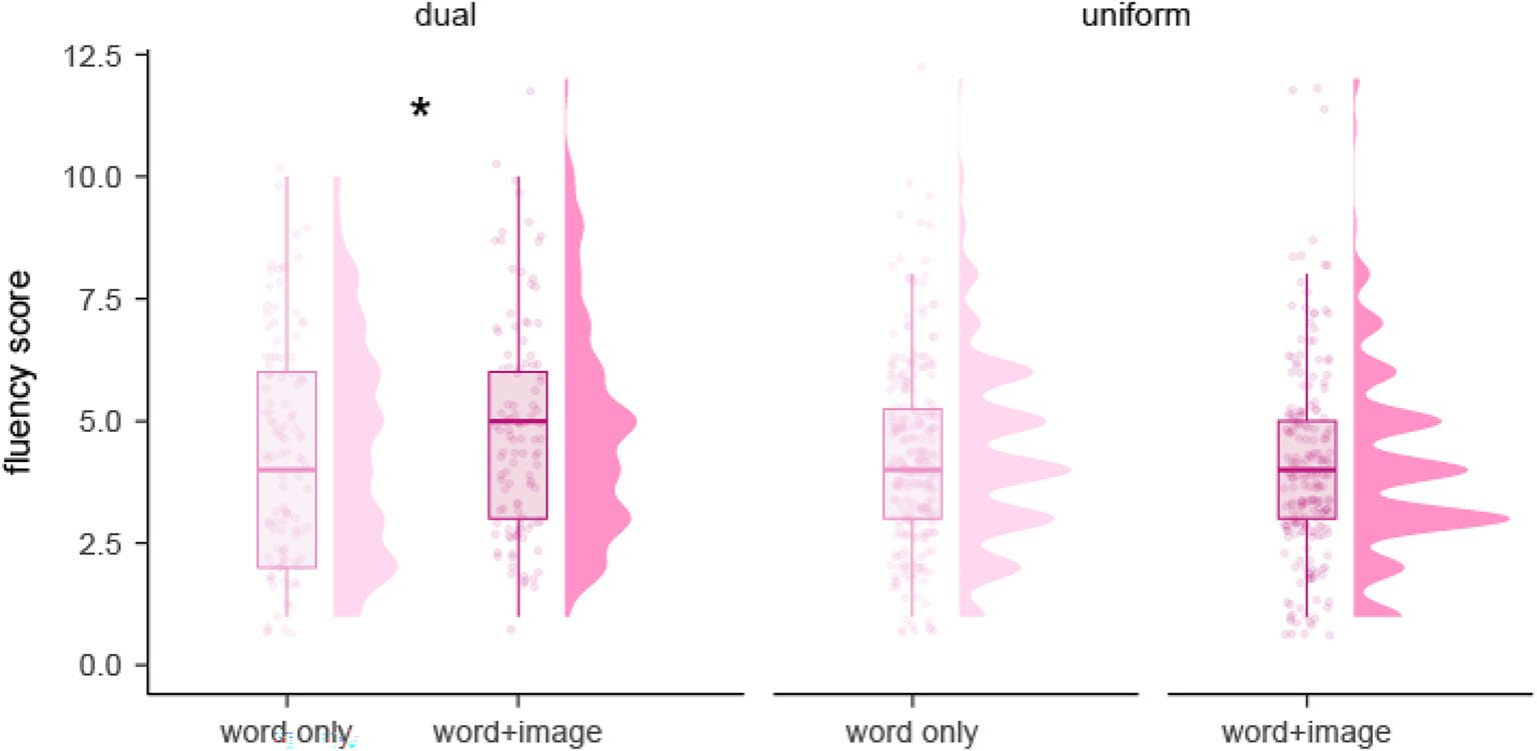
Idea fluency distribution in the three experimental procedures: dual (within-subject), uniform: word only (between-subject), and uniform: word + image (between-subject).

As regards idea fluency, we found a marginally significant effect of condition in the dual procedure (within-subject comparison), with higher number of ideas generated for word + image than word only items (*p* = *0.051, η*_*p*_^*2*^ = *0.144,* large effect size*;* Supplementary Table 1, Fig. 1).

When comparing between participants in the uniform procedures, we did not find significant differences in idea fluency between conditions (*p* = 0.448, *η*_*p*_^*2*^ = *0.013*; Supplementary Table 2). When comparing across uniform procedures within each condition, we did not find any significant difference either, be it in the word only condition (*p* = 0.757, *η*_*p*_^*2*^ = *0.002*) or the word + image condition (*p* = 0.071, *η*_*p*_^*2*^ = *0.067,* medium effect size; Supplementary Table 3 and 4).

**Figure 2:**
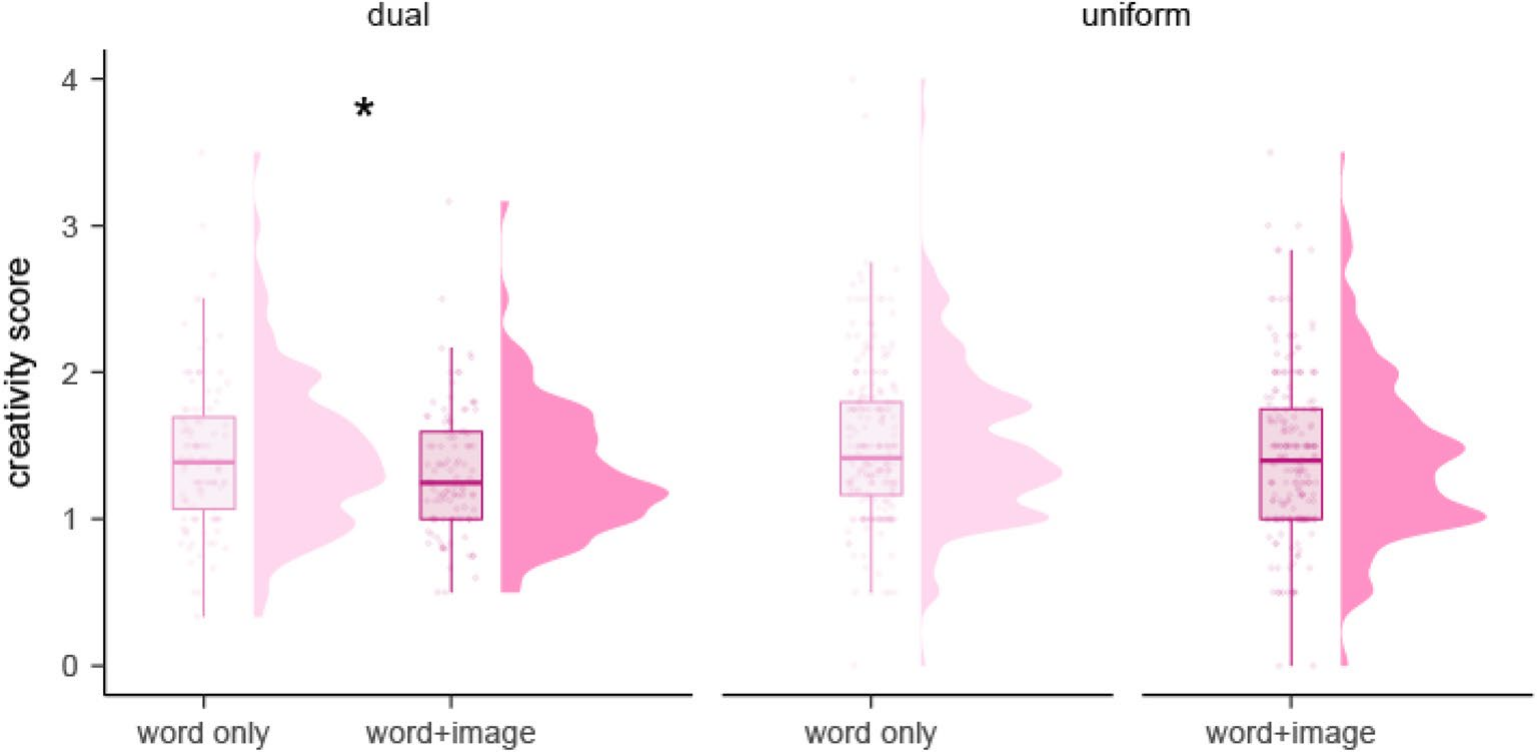
Distribution of creativity ratings in the three procedures of Experiment 1: Dual procedure: word only and word + image conditions in the same participants, uniform procedures: word only or word + image, in different participants.

Turning to idea creativity, we found a significant fixed effect of condition in the dual procedure (*p* = 0.048, *η*_*p*_^*2*^ = *0.146,* large effect size), with lower creativity scores in word + image (*M* = 1.32, 95% CI [1.26, 1.38]) than word only trials (*M* = 1.41, 95% CI [1.34, 1.48]; Supplementary Table 5). We also found that variance in creativity dropped significantly for word + image items (σ^2^ = 0.50) as compared with word only items (σ^2^ = 0.59; *F*(522,483) = 0.86, *p* = 0.043; Fig. 2).

When comparing word only and word + image conditions across uniform procedures (between-subject comparison), there was no significant difference (*p* = . 189, *η*_*p*_^*2*^ = *0.034;* Supplementary Table 6) and variance did not differ between conditions either; *F*(1001,817) = 1.03, *p* = 0.666.

When considering differences between procedures, there was no significant difference for the word only condition (*p* = 0.221, *η*_*p*_^*2*^ = *0.034*) or the word + image condition (*p* = 0.362, *η*_*p*_^*2*^ = *0.019*; Supplementary Table 7 and 8).

### Experiment 2: AUT procedure in the list and cycle format

**Figure 3:**
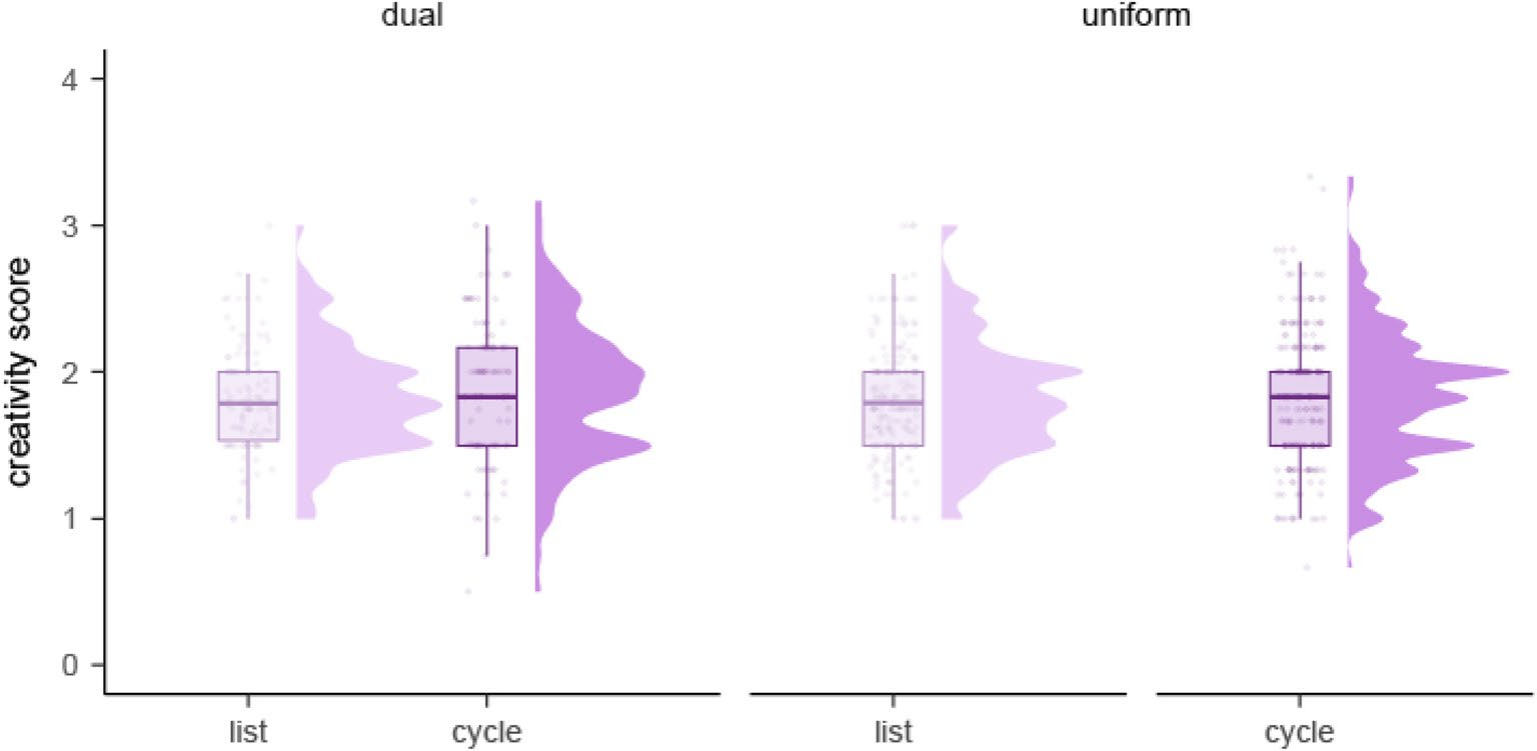
Distribution of creativity ratings in the three versions of Experiment 2: list, cycle, and dual (within-subject).

In Experiment 2, we found no significant difference in creativity overall between the list and cycle conditions of the dual procedure (within-subject comparison; *p* = 0.118, *η*_*p*_^*2*^ = *0.089*; Supplementary Table 9; Fig. 3).

**Figure 4:**
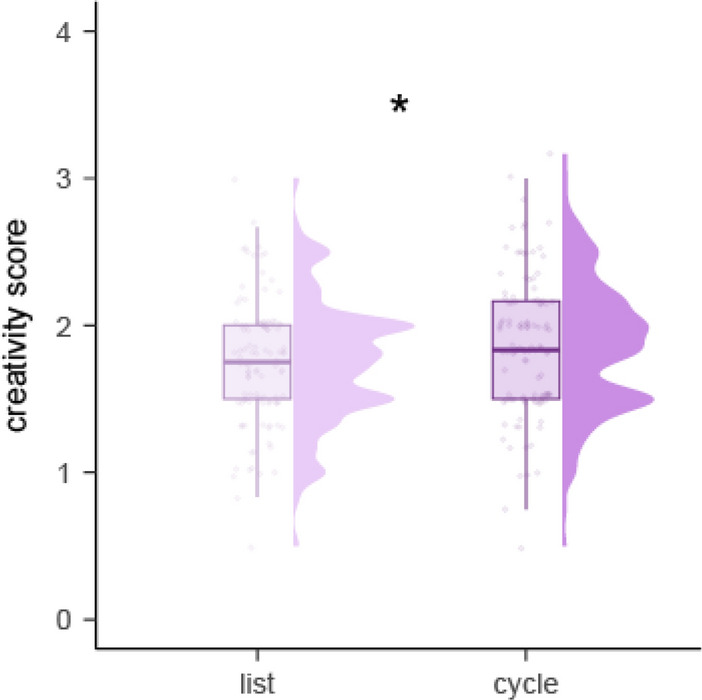
Distribution of creativity ratings for the first three ideas generated in the list and cycle conditions of the dual procedure (within-subject) of Experiment 2.

Because participants could only generate one idea per cycle in the cycle condition, we also compared the first three ideas generated in the list condition with the three ideas generated in the cycle condition in the dual procedure. We found a significant fixed effect of condition, with more creative ideas in the cycle (*M* = 1.89, 95% CI [1.82, 1.96]) than the list (*M* = 1.73, 95% CI [1.66, 1.80]) condition (*p* = 0.026, *η*_*p*_^*2*^ = *0.165,* large effect size; Supplementary Table 10 and Fig. 4).

When comparing creative ratings between participants across the uniform procedures, there was no significant difference between the list and cycle conditions (*p* = 0.721, *η*_*p*_^*2*^ = *0.003*; Supplementary Table 11).

**Figure 5:**
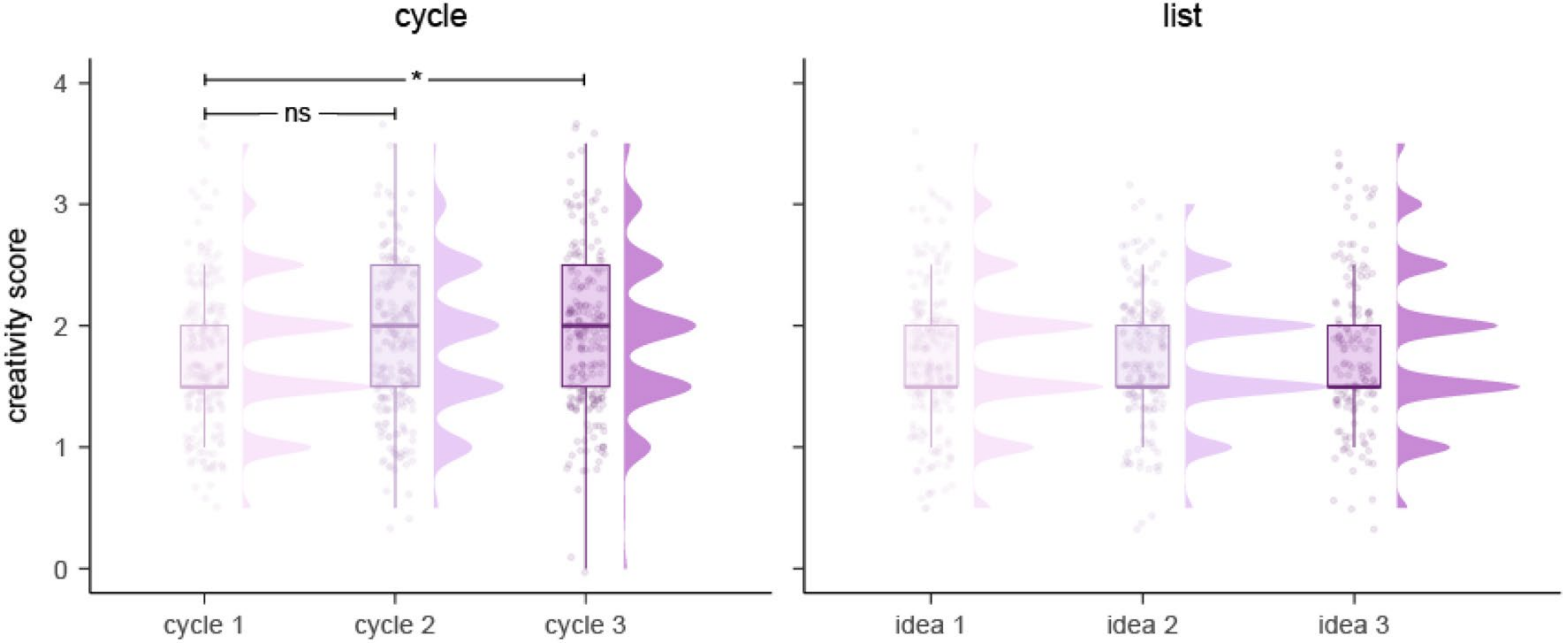
Creativity ratings generated in for the first three ideas in the cycle and list procedure in Experiment 2.

When comparing creativity ratings for the first three ideas in the cycle and list procedure, we found a significant effect of idea sequence in the cycle (*p* = 0.016) but not the list (*p* = 0.081) procedure (Fig. 5, Supplementary Table 12 and 13). Post hoc pairwise comparisons revealed significantly higher creativity ratings for ideas generated in cycle three (*M* = 1.91, 95% CI [1.77, 2.05]) than cycle one (*M* = 1.76, 95% CI [1.63, 1.90]), *t*(589.89) = -2.78, SE = 0.05, *p* = 0.015.

When considering differences across the dual and uniform procedures, there was no significant difference either in the list (*p* = 0.979, *η*_*p*_^*2*^ =  < *0.001*) or cycle (*p* = 0.545, *η*_*p*_^*2*^ = *0.007*) conditions (*p*_s_ > 0.1; Supplementary Table 14 and 15).

## Methods

### Experiment 1: AUT items as (written) word or word + image

#### Participants

Sixty-seven participants (*M*_*age*_ = 21, *min*_*age*_ = 18, *max*_*age*_ = 37; 48 women, 14 men, 5 nonbinary) gave informed consent to take part in the experiment. All participants were Polish native speakers, they had normal or corrected-to-normal vision and hearing, and no neurological or language-related disorders.

#### Stimuli

Thirty everyday objects were selected from previous studies on the AUT to be presented either as word only or word + image (image overlaid with a word). Item number was in the middle of the range used in the literature (one^19^ to 60^29^), so as to allow item variation between participants (ten items randomly selected for each participant) and measure inter-item variability in our analyses.

Words, mostly selected from previous publications using the AUT as the core task, were presented in Arial font, in size 28 (for word + image stimuli) or 32 (for word stimuli) points and 2-point character spacing, in white outlined in black (for word + image stimuli only, text glow option in PowerPoint, to maximise readability when superimposed on images in the word + image condition). Stimuli are listed on the Open Science Framework server (link in the Data availability statement).

**Figure 6:**
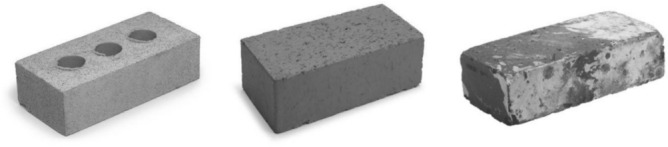
Three pictures of bricks used in the norming procedure.

For each item, three images deemed “a standard depiction” by all researchers in our group were collated based on Google image searches/BOSS^35^ and Linguapix^36^ databases. Items were then presented within two norming sessions to 20 (27 items) and 24 participants (5 items) participants. Participants (1) provided the first label that came to mind upon viewing each image presented in isolation, (2) selected one photo out of three that in their opinion best depicted the object keyword, and (3) rated the degree of relatedness between each picture and word on a scale of 0 to 4 (see Supplementary Table 16; Fig. 6).

#### Procedure

Participants were randomly assigned to one of three experimental procedures: (i) uniform with word only condition items (*n* = 24), (ii) uniform with word + image condition items (image overlaid with a word, *n* = 21), or (iii) dual, consisting of half with word only and half with word + image trials (*n* = 22). Disparity in counts of occurrence is due to random allocation of procedures to participants. Each experimental procedure featured ten items selected randomly from a pool of 30. In the word only condition, items were single words presented at the centre of the screen. In the word + image condition, the same words were presented with their best matching image selected from norming, as a backdrop. The dual procedure was implemented to increase statistical power (within-subject comparisons) and investigate possible contextual effects of one condition on the other.

All procedures had the same internal structure. On each trial, a randomly selected AUT item was presented at the centre of the screen. Images were presented as background to words on a 23-inch LCD monitor with a resolution of 1920 × 1080. Selection was random without repetition (i.e., no item could be selected both as word only and word + image). When prompted, participants had 15 s to type in a common use for that item. Next, participants had two minutes to type in unusual yet possible uses for that item. In the dual procedure, items were blocked (word only, image + word) with block order counterbalanced between participants. Participants completed the experiment in about 30 min and received 50 PLN or credit points for taking part in the experiment.

#### Behavioural data analysis

Data preprocessing was done using the *tidyverse* package^37^ in R^38^. Participants’ creativity scores were analysed with linear-mixed models (LMM) using *lme4*^39^ and *lmerTest* packages^40^. Tables with model summaries were computed with the *sjPlot* package^41^. Estimates of effect size were calculated using the *effectsize* package^42^.

For each LMM, we first computed a maximal model with a full random-effect structure, including subject- and item-related variance components for intercepts and by-subject and by-item random slopes for fixed effects^43^. All fixed effects were coded using sum contrast coding. If maximal models did not converge and/or turned out to be too complex, we progressively selected more parsimonious LMMs following the recommendations from Bates, Kliegl, Vasishth, and Baayen (2018)^44,45^. Small variance parameters were removed using the *lme4::rePCA* and *lme4::VarCorr*^46^ functions until the LMMs were supported by the data. The final structure of each model is presented below:

Idea fluency:

1. Dual procedure (within-subject), word only vs. word + image: *fluency* ~ *word_only_vs_word_plus_image* + *(1* + *word_only_vs_word_plus_image|participant)* + *(1|item)*
2. Uniform procedure (between-subject), word only vs. word + image: *fluency* ~ *word_only_vs_word_plus_image* + *(1|participant)* + *(1|item)*
3. Word only condition, uniform vs. dual procedure (between-subject): *fluency* ~ *uniform_vs_dual* + *(1|participant)* + *(1|item)*
4. Word + image condition, uniform vs. dual procedure (between-subject): *fluency* ~ *uniform_vs_dual* + *(1|participant)* + *(1* + *uniform_vs_dual|item)*

Idea creativity:

1. Dual procedure (within-subject), word only vs. word + image: *Rating* ~ *word_only_vs._word_plus_image* + *(1* + *word_only_vs._word_plus_image|participant)* + *(1|item)*
2. Uniform procedure (between-subject), word only vs. word + image: *Rating* ~ *word_only_vs._word_plus_image* + *(1|participant)* + *(1* + *word_only_vs_word_plus_image|item)*
3. Word only condition, uniform vs. dual procedure (between-subject): *Rating* ~ *uniform_vs._dual* + *(1|participant)* + *(1* + *uniform_vs._dual|item)*
4. Word + image condition, uniform vs. dual procedure (between-subject): *Rating* ~ *uniform_vs_dual* + *(1|participant)* + *(1|item)*

Estimates and significance of fixed effects and interactions (*p*-values) are based on the Satterthwaite approximation for LMM.

#### Assessment of AUT performance

Originality ratings were compiled and processed following the Consensual Assessment Technique (CAT)^47–50^. Four raters judged the creativity of ideas produced by participants on a 5-point Likert scale (0—common, unoriginal use, 1—not very original but a bit uncommon, 2—quite original and quite uncommon, 3—original, uncommon, 4—highly original, rare). Raters were instructed that an answer considered creative needed to be novel and/or unique whilst being principally possible; principally impossible uses were given a score of 10 and were excluded from further analyses. For details about instructions for raters, see Supplementary Information. Raters were also encouraged to use the full-scale range. Inter-rater reliability was very high [ICC(C,4) = 0.93]. Ratings were averaged across raters, resulting in one creativity rating per item and participant.

### Experiment 2: AUT procedure as list or cycle

#### Participants

Seventy-two participants (*M*_*age*_ = 22, *min*_*age*_ = 18, *max*_*age*_ = 41; 49 women, 14 men, 8 nonbinary) gave informed consent to take part in the experiment. All participants were Polish native speakers, they had normal or corrected-to-normal vision and hearing, and no neurological or language-related disorders.

#### Stimuli

Stimuli were identical to the word + image stimuli described in Experiment 1.

#### Procedure

Participants were randomly assigned to one of three experimental procedures: (i) uniform with list trials (*n* = 22), (ii) uniform with cycle trials (*n* = 25), or dual (half list and half cycle trials, *n* = 24), disparity between counts being due to random allocation of procedures to participants. Each experimental procedure featured ten items selected randomly from a pool of 30 items and presented as word + image in all cases. In the list condition, participants had two minutes to list unusual uses for ten randomly selected AUT items. In the cycle condition, participants engaged in three successive 30 s cycles of idea generation-evaluation-selection per item. In each cycle, they were asked to think of unusual uses for the current item displayed at the beginning of each cycle and report their *single best* idea by typing into a blue frame displayed at the end of the cycle, so as to foster evaluation. In the dual procedure, participants engaged in a block of five list trials and a block of five cycle trials (with items randomly selected without repetition), and block order was counterbalanced between participants.

In all experimental procedures, participants first saw a randomly selected AUT item and typed in its common use (within 15 s). Next, they were presented with the item again and asked to come up with unusual yet possible uses for each object (within a list or cycle context). Thus, apart from the registration of the common use, the same item was presented three times in a row in the cycle but only once in the list condition. Participants completed the experiment in about 30 min. They received 50 PLN or credit points for taking part in the experiment.

#### Behavioural data analysis

Preprocessing and data analysis followed the same approach as in Experiment 1. The final structure of each model in Experiment 2 is presented below.

1. Dual procedure (within-subject), list vs. cycle condition:
2. *Rating* ~ *list_vs_cycle* + *(1* + *list_vs_cycle|participant)* + *(1|item)*
3. Uniform procedures, list vs. cycle condition (between-subject): *Rating* ~ *list_vs_cycle* + *(1|participant)* + *(1* + *list_vs_cycle|item)*
4. Cycle condition, uniform vs. dual procedure (between-subject): *Rating* ~ *uniform_vs_dual* + *(1|participant)* + *(1|item)*
5. List condition, uniform vs. dual procedure (between-subject): *Rating* ~ *uniform_vs_dual* + *(1|participant)* + *(1|item)*

#### Assessment of AUT performance

Three raters evaluated the creativity of ideas produced by participants following the same protocol as in Experiment 1. Inter-rater reliability was very high (ICC(C,3) = 0.91).

#### Ethics statement

The study was designed and performed in accordance with the Declaration of Helsinki. The Ethics Committee for Research Involving Human Participants at Adam Mickiewicz University, Poznań approved the study (Resolution no. 23/2021/2022). Participants were informed about the study protocol and gave their informed consent to participate in the study.

## Discussion

In two experiments, we set out to test for differences in the AUT (i) elicited by words presented in isolation or together with an image, and (ii) related to the format of idea production, list or cycle.

In Experiment 1, the picture was used to disambiguate the word in word + image trials. Whilst fluency was greater in the word + image condition, we found the expected slight drop in originality ratings for word + image compared to word only trials. We also found the expected lower variance in originality ratings for word + image than word only prompts. In both cases, however, the differences were only found in the within-subject, not the between-subject comparisons.

In Experiment 2, as expected we found that creativity was significantly higher in the cycle than list condition, albeit only when the output was quantitatively matched, that is, for the first three ideas of the list condition. Furthermore, idea creativity increased with cycle, although a significant difference was only found between cycle 3 and cycle 1. We found no equivalent difference in the list condition. Interestingly, as was the case in Experiment 1, both differences found here concerned within-subject comparisons, not between-subject comparisons.

As mentioned in the introduction, Chrysikou et al.^28^ and George et al.^29^ showed that inclusion of images in the AUT can affect the quality of generated ideas, resulting in normative and less original answers. However, one should not deduct from this result, which is consistent with ours in Experiment 1, that introducing images alongside words is undesirable (e.g., because it reduces idea originality at face value). Indeed, the relatively lower originality measured in the word + image condition is likely linked to reduced variability between participants, since the disambiguating picture would have harmonised participant expectations regarding the objects referred to by the prompts. However, we note that idea fluency was significantly greater in the image + word condition than the word only condition, which can be seen as a trade-off effect (more productive creativity in the image + word condition). This observation might relate to the increased level of constraint realised by the introduction of a disambiguating picture. Haught^51^ showed that presenting a picture as a prompt led to more creativity in a sentence production task than presenting words, in line with predictions of the Integrated Constrains in Creativity model^52^. Taken together, the disambiguating effect of images and the implicit constraint imposed by them may have compensated each other to result in only mild differences between the two experimental conditions in Experiment 1.

A review of the literature on the AUT as a classic paradigm to quantify creativity shows that most studies used word only prompts^6,18–22^ which we believe might have inflated inter-individual variability at the outset of each trial, due to the lack of specification inherent to word labels. Thus, if images appear to lower originality in the AUT, this may be a necessary sacrifice to homogenise the conceptual plane from which participants start ideation. Furthermore, it must be noted that improving the baseline not only benefits participant data but also enhances the reliability of the rating procedure. If, as participants do, raters start from a common ground when being exposed to images as well as labels, they are more likely to deliver consistent ratings. The effect of presenting images together with words thus contributes to reducing spurious variability (that can be mistaken for originality) on both the level of participants and raters.

In Experiment 2, we attempted to increase the contribution of evaluation in order to enhance the potency of the AUT to measure creativity as opposed to merely idea originality. As expected, we found that the first three ideas of the list procedure were less original than the three ideas generated during the cycle procedure. This observation is *convergent* with the serial order effect often reported in AUT, which has been linked to progressively greater involvement of executive function as time passes on task and resulting in greater originality for ideas generated later^19^. This is further compatible with the observation of greater originality in cycle 3 compared to cycle 1 observed here, which can be construed as a serial order effect across cycles in our adaptation of the task. These results are also consistent with the idea that increasing the level of constraint in a creative idea generation task like the AUT leads to greater idea originality^52^. We note, however, that this difference did not apply overall, which also suggests, *a contrario*, that the originality of ideas produced in the list procedure did not significantly go beyond that of the cycle procedure. Thus, the cycle procedure appears to have captured most of the originality to be expected in the AUT in conditions where participants are more readily comparable with one another, because the number of items is constant.

In both experiments, between-subject comparisons yielded almost no difference between conditions, and this is presumably due to uncontrolled variance stemming from individual differences and likely superseding subtle effects that are detected in within-subject comparisons. We thus argue that measures of creativity using the AUT as the core paradigm should be compared within-subject wherever possible. Furthermore, we acknowledge both widely recognised limitations of the AUT as a tool to measure creative ideation and more subtle limitations relating to the kind of cognitive processes at work in this task that significantly depart from what can be expected in more ecologically valid contexts. First, we acknowledge that the AUT is a mostly artificial task since people are rarely asked to come up with as many creative uses for an object as possible within a limited time period (except perhaps in engineering design education). However, our goal was not to propose a more ecologically valid adaptation of the AUT. It was rather to improve the baseline from which participants start and increase the evaluative dimension of the task. This was made possible by comparing our manipulations to standard AUT measures established in the field.

In conclusion, using pictures in combination with words may disambiguate the cue in tasks measuring creativity and participants’ performance may be more comparable across conditions, whilst incurring minimal loss of originality. This is even more important considering that the same manipulation is likely to have had the same disambiguating effect on raters, and may thus have contributed to the high inter-rater reliability in our experiments. We believe that the cycle procedure employed here is a valid way of fostering idea evaluation without incurring a loss of originality whilst making participants’ output more comparable than that of a list procedure. Finally, the lack of difference observed here in between-group comparisons argues in favour of using within-subject designs whenever possible. Although this is true of essentially all comparisons in experimental psychology, this seems to be particularly relevant in the domain of creativity testing.

### Supplementary Information

Supplementary Information.

## Data Availability

Stimuli, raw data, ratings, and R scripts are listed on the Open Science Framework server (https://osf.io/q5hfz/?view_only=c4e85e49224848f5bd56f0d4949521ba).
