# Supplementary material for: Controlling stimulus ambiguity reduces spurious creative ideation variance in a cyclic adaptation of the alternative uses task
Source: Sci Rep. 2024 May 31;14:12492. doi: 10.1038/s41598-024-63225-2 (PMC11143269; doi:10.1038/s41598-024-63225-2)
Supplement: Supplementary file 1 — Supplementary Information. [file 41598_2024_63225_MOESM1_ESM.docx]

# Supplementary Tables

Supplementary Table 1. Summary table for the within-subject LME model comparing idea fluency scores in the word only vs. word+image condition of the dual version of Experiment 1.

| *Predictors* | *Estimates* | *std. Error* | *CI* | *p* | *Eta-squared (partial)* |
| --- | --- | --- | --- | --- | --- |
| (Intercept) | 4.67 | 0.36 | 3.95 – 5.38 | **<0.001** |  |
| word only vs word+image | -0.45 | 0.23 | -0.90 – 0.00 | 0.051 | 0.144 |
| **Random Effects** | | | | |  |
| σ^2f^ | 2.26 | | | |  |
| τ_00_ _item_ | 0.35 | | | |  |
| τ_00_ _participant_ | 2.42 | | | |  |
| τ_11_ _participant.word only_vs_word+image_ | 0.17 | | | |  |
| ρ_01_ _participant_ | 0.16 | | | |  |
| ICC | 0.56 | | | |  |
| N _participant_ | 22 | | | |  |
| N _item_ | 30 | | | |  |
| Observations | 220 | | | |  |
| Marginal R^2^ / Conditional R^2^ | 0.010 / 0.559 | | | |  |

Supplementary Table 2. Summary table for the between-subject LME model comparing idea fluency scores from the uniform word only and word+image versions of Experiment 1.

| *Predictors* | *Estimates* | *std. Error* | *CI* | *p* | *Eta-squared (partial)* |
| --- | --- | --- | --- | --- | --- |
| (Intercept) | 4.18 | 0.23 | 3.72 – 4.63 | **<0.001** |  |
| word only vs word+image | 0.30 | 0.39 | -0.48 – 1.07 | 0.448 | 0.013 |
| **Random Effects** | | | | |  |
| σ^2^ | 1.62 | | | |  |
| τ_00_ _participant_ | 1.57 | | | |  |
| τ_00_ _item_ | 0.44 | | | |  |
| ICC | 0.55 | | | |  |
| N _participant_ | 45 | | | |  |
| N _item_ | 30 | | | |  |
| Observations | 449 | | | |  |
| Marginal R^2^ / Conditional R^2^ | 0.006 / 0.556 | | | |  |

Supplementary Table 3. Summary table for the between-subject LME model comparing idea fluency scores from the word only condition in participants performing the uniform and dual versions of Experiment 1.

| *Predictors* | *Estimates* | *std. Error* | *CI* | *p* | *Eta-squared (partial)* |
| --- | --- | --- | --- | --- | --- |
| (Intercept) | 4.43 | 0.27 | 3.89 – 4.97 | **<0.001** |  |
| word only uniform vs dual | -0.14 | 0.45 | -1.03 – 0.75 | 0.757 | 0.002 |
| **Random Effects** | | | | |  |
| σ^2^ | 1.68 | | | |  |
| τ_00_ _participant_ | 2.06 | | | |  |
| τ_00_ _item_ | 0.42 | | | |  |
| ICC | 0.60 | | | |  |
| N _participant_ | 46 | | | |  |
| N _item_ | 30 | | | |  |
| Observations | 350 | | | |  |
| Marginal R^2^ / Conditional R^2^ | 0.001 / 0.597 | | | |  |

Supplementary Table 4. Summary table for the between-subject LME model comparing idea fluency scores from the word+image condition in participants performing the uniform and dual versions of Experiment 1.

| *Predictors* | *Estimates* | *std. Error* | *CI* | *p* | *Eta-squared (partial)* |
| --- | --- | --- | --- | --- | --- |
| (Intercept) | 4.60 | 0.26 | 4.10 – 5.11 | **<0.001** |  |
| word+image uniform vs. dual | -0.85 | 0.47 | -1.78 – 0.07 | 0.071 | 0.067 |
| **Random Effects** | | | | |  |
| σ^2^ | 1.88 | | | |  |
| τ_00_ _participant_ | 1.95 | | | |  |
| τ_00_ _item_ | 0.24 | | | |  |
| τ_11_ _item.word+image uniform_vs. dual_ | 0.40 | | | |  |
| ρ_01_ _item_ | 0.50 | | | |  |
| ICC | 0.56 | | | |  |
| N _participant_ | 43 | | | |  |
| N _item_ | 30 | | | |  |
| Observations | 319 | | | |  |
| Marginal R^2^ / Conditional R^2^ | 0.037 / 0.580 | | | |  |

Supplementary Table 5. Summary table for the within-subject LME model comparing creativity scores in the word only vs. word+image condition of the dual version of Experiment 1.

| *Predictors* | *Estimates* | *std. Error* | *CI* | *p* | *Eta-squared (partial)* |
| --- | --- | --- | --- | --- | --- |
| (Intercept) | 1.37 | 0.05 | 1.26 – 1.47 | **<0.001** |  |
| word only vs word+image | 0.10 | 0.05 | 0.00 – 0.20 | **0.048** | 0.146 |
| **Random Effects** | | | | |  |
| σ^2^ | 0.49 | | | |  |
| τ_00_ _item_ | 0.01 | | | |  |
| τ_00_ _participant_ | 0.05 | | | |  |
| τ_11_ _participant.word only_vs._word+image_ | 0.01 | | | |  |
| ρ_01_ _participant_ | 0.12 | | | |  |
| ICC | 0.11 | | | |  |
| N _participant_ | 22 | | | |  |
| N _item_ | 30 | | | |  |
| Observations | 1007 | | | |  |
| Marginal R^2^ / Conditional R^2^ | 0.005 / 0.111 | | | |  |

Supplementary Table 6. Summary table for the between-subject LME model comparing creativity scores from the uniform word only and word+image versions of Experiment 1.

| *Predictors* | *Estimates* | *std. Error* | *CI* | *p* | *eta-squared (partial)* |
| --- | --- | --- | --- | --- | --- |
| (Intercept) | 1.45 | 0.05 | 1.36 – 1.55 | **<0.001** |  |
| word only vs word+image | 0.12 | 0.09 | -0.06 – 0.30 | 0.189 | 0.034 |
| **Random Effects** | | | | |  |
| σ^2^ | 0.53 | | | |  |
| τ_00_ _participant_ | 0.07 | | | |  |
| τ_00_ _item_ | 0.01 | | | |  |
| τ_11_ _item.word only_vs_word+image_ | 0.03 | | | |  |
| ρ_01_ _item_ | 0.05 | | | |  |
| ICC | 0.14 | | | |  |
| N _participant_ | 45 | | | |  |
| N _item_ | 30 | | | |  |
| Observations | 1820 | | | |  |
| Marginal R^2^ / Conditional R^2^ | 0.006 / 0.146 | | | |  |

Supplementary Table 7. Summary table for the between-subject LME model comparing creativity scores from the word only condition in participants performing the uniform and dual versions of Experiment 1.

| *Predictors* | *Estimates* | *std. Error* | *CI* | *p* | *Eta-squared (partial)* |
| --- | --- | --- | --- | --- | --- |
| (Intercept) | 1.45 | 0.05 | 1.35 – 1.54 | **<0.001** |  |
| word only uniform vs. dual | 0.10 | 0.08 | -0.06 – 0.27 | 0.221 | 0.034 |
| **Random Effects** | | | | |  |
| σ^2^ | 0.54 | | | |  |
| τ_00_ _participant_ | 0.06 | | | |  |
| τ_00_ _item_ | 0.01 | | | |  |
| τ_11_ _item.word only uniform_vs. dual_ | 0.01 | | | |  |
| ρ_01_ _item_ | 0.78 | | | |  |
| ICC | 0.12 | | | |  |
| N _participant_ | 46 | | | |  |
| N _item_ | 30 | | | |  |
| Observations | 1486 | | | |  |
| Marginal R^2^ / Conditional R^2^ | 0.004 / 0.124 | | | |  |

Supplementary Table 8. Summary table for the between-subject LME model comparing creativity scores from the word+image condition in the uniform vs. dual versions of Experiment 1.

| *Predictors* | *Estimates* | *std. Error* | *CI* | *p* | *Eta-squared (partial)* |
| --- | --- | --- | --- | --- | --- |
| (Intercept) | 1.35 | 0.05 | 1.25 – 1.45 | **<0.001** |  |
| word+image uniform vs. dual | 0.08 | 0.09 | -0.09 – 0.25 | 0.362 | 0.019 |
| **Random Effects** | | | | |  |
| σ^2^ | 0.49 | | | |  |
| τ_00_ _participant_ | 0.06 | | | |  |
| τ_00_ _item_ | 0.02 | | | |  |
| ICC | 0.14 | | | |  |
| N _participant_ | 43 | | | |  |
| N _item_ | 30 | | | |  |
| Observations | 1341 | | | |  |
| Marginal R^2^ / Conditional R^2^ | 0.003 / 0.143 | | | |  |

Supplementary Table 9. Summary table for the within-subject LME model comparing creativity scores in the cycle vs. list procedure of the dual version of Experiment 2.

| *Predictors* | *Estimates* | *std. Error* | *CI* | *p* | *Eta-squared (partial)* |
| --- | --- | --- | --- | --- | --- |
| (Intercept) | 1.85 | 0.06 | 1.73 – 1.96 | **<0.001** |  |
| cycle vs list | -0.09 | 0.06 | -0.20 – 0.02 | 0.118 | 0.089 |
| **Random Effects** | | | | |  |
| σ^2^ | 0.33 | | | |  |
| τ_00_ _stimulus_ | 0.02 | | | |  |
| τ_00_ _participant_ | 0.04 | | | |  |
| τ_11_ _participant.cycle_vs_list_ | 0.02 | | | |  |
| ρ_01_ _participant_ | -0.76 | | | |  |
| ICC | 0.17 | | | |  |
| N _participant_ | 22 | | | |  |
| N _stimulus_ | 30 | | | |  |
| Observations | 741 | | | |  |
| Marginal R^2^ / Conditional R^2^ | 0.005 / 0.169 | | | |  |

Supplementary Table 10. Summary table for another within-subject LME model comparing creativity scores for the first three ideas generated in the cycle vs. list conditions of the dual version of Experiment 2.

| *Predictors* | *Estimates* | *std. Error* | *CI* | *p* | *Eta-squared (partial)* |
| --- | --- | --- | --- | --- | --- |
| (Intercept) | 1.81 | 0.06 | 1.70 – 1.93 | **<0.001** |  |
| cycle vs. list | -0.14 | 0.06 | -0.27 – -0.02 | **0.026** | 0.165 |
| **Random Effects** | | | | |  |
| σ^2^ | 0.31 | | | |  |
| τ_00_ _stimulus_ | 0.04 | | | |  |
| τ_00_ _participant_ | 0.03 | | | |  |
| τ_11_ _participant._ _cycle vs. list_ | 0.03 | | | |  |
| ρ_01_ _participant_ | -0.76 | | | |  |
| ICC | 0.20 | | | |  |
| N _participant_ | 22 | | | |  |
| N _stimulus_ | 30 | | | |  |
| Observations | 583 | | | |  |
| Marginal R^2^ / Conditional R^2^ | 0.013 / 0.213 | | | |  |

Supplementary Table 11. Summary table for the between-subject LME model comparing creativity scores from the uniform cycle vs. list versions of Experiment 1.

| *Predictors* | *Estimates* | *std. Error* | *CI* | *p* | *Eta-squared (partial)* |
| --- | --- | --- | --- | --- | --- |
| (Intercept) | 1.83 | 0.05 | 1.74 – 1.91 | **<0.001** |  |
| cycle vs. list | -0.03 | 0.07 | -0.16 – 0.11 | 0.721 | 0.003 |
| **Random Effects** | | | | |  |
| σ^2^ | 0.30 | | | |  |
| τ_00_ _participant_ | 0.04 | | | |  |
| τ_00_ _stimulus_ | 0.03 | | | |  |
| τ_11_ _stimulus._ _cycle vs. list_ | 0.00 | | | |  |
| ρ_01_ _stimulus_ | 0.84 | | | |  |
| ICC | 0.19 | | | |  |
| N _participant_ | 44 | | | |  |
| N _stimulus_ | 30 | | | |  |
| Observations | 1459 | | | |  |
| Marginal R^2^ / Conditional R^2^ | 0.000 / 0.192 | | | |  |

*Supplementary Table 12. Summary table for the within-subject LME model comparing creativity scores across cycles in the cyclic version of Experiment 2.*

| *Predictors* | *Estimates* | *std. Error* | *CI* | *p* | *Eta-squared (partial)* |
| --- | --- | --- | --- | --- | --- |
| (Intercept) | 1.84 | 0.06 | 1.72 – 1.95 | **<0.001** |  |
| Idea1 | -0.00 | 0.06 | -0.12 – 0.12 | 0.998 |  |
| Idea2 | -0.15 | 0.06 | -0.27 – -0.03 | **0.016** |  |
| **Random Effects** | | | | |  |
| σ^2^ | 0.29 | | | |  |
| τ_00_ _stimulus_ | 0.02 | | | |  |
| τ_00_ _participant_ | 0.06 | | | |  |
| ICC | 0.21 | | | |  |
| N _participant_ | 24 | | | |  |
| N _stimulus_ | 30 | | | |  |
| Observations | 637 | | | |  |
| Marginal R^2^ / Conditional R^2^ | 0.010 / 0.220 | | | |  |

Supplementary Table 13. Summary table for the within-subject LME model comparing creativity scores across the first three ideas in the list version of Experiment 2.

| *Predictors* | *Estimates* | *std. Error* | *CI* | *p* | *Eta-squared (partial)* |
| --- | --- | --- | --- | --- | --- |
| (Intercept) | 1.79 | 0.06 | 1.68 – 1.90 | **<0.001** |  |
| Idea1 | 0.03 | 0.07 | -0.11 – 0.17 | 0.648 |  |
| Idea2 | -0.13 | 0.07 | -0.28 – 0.02 | 0.081 |  |
| **Random Effects** | | | | |  |
| σ^2^ | 0.28 | | | |  |
| τ_00_ _stimulus_ | 0.04 | | | |  |
| τ_00_ _participant_ | 0.03 | | | |  |
| ICC | 0.19 | | | |  |
| N _participant_ | 20 | | | |  |
| N _stimulus_ | 30 | | | |  |
| Observations | 516 | | | |  |
| (Intercept) | 1.79 | | | | 0.06 |

Supplementary Table 14. Summary table for the between-subject LME model comparing creativity scores from the cycle procedure in participants performing the cycle and dual versions of Experiment 2.

| *Predictors* | *Estimates* | *std. Error* | *CI* | *p* | *Eta-squared (partial)* |
| --- | --- | --- | --- | --- | --- |
| (Intercept) | 1.87 | 0.05 | 1.77 – 1.98 | **<0.001** |  |
| uniform vs. dual | 0.05 | 0.08 | -0.11 – 0.21 | 0.542 | 0.008 |
| **Random Effects** | | | | |  |
| σ^2^ | 0.31 | | | |  |
| τ_00_ _participant_ | 0.06 | | | |  |
| τ_00_ _stimulus_ | 0.02 | | | |  |
| ICC | 0.21 | | | |  |
| N _participant_ | 46 | | | |  |
| N _stimulus_ | 30 | | | |  |
| Observations | 942 | | | |  |
| Marginal R^2^ / Conditional R^2^ | 0.001 / 0.210 | | | |  |

Supplementary Table 15. Summary table for the between-subject LME model comparing creativity scores from the list procedure in participants performing the list and dual versions of Experiment 2.

| *Predictors* | *Estimates* | *std. Error* | *CI* | *p* | *Eta-squared (partial)* |
| --- | --- | --- | --- | --- | --- |
| (Intercept) | 1.81 | 0.05 | 1.72 – 1.90 | **<0.001** |  |
| uniform vs. dual | 0.00 | 0.06 | -0.12 – 0.12 | 0.979 | < 0.001 |
| **Random Effects** | | | | |  |
| σ^2^ | 0.30 | | | |  |
| τ_00_ _participant_ | 0.02 | | | |  |
| τ_00_ _stimulus_ | 0.03 | | | |  |
| ICC | 0.15 | | | |  |
| N _participant_ | 42 | | | |  |
| N _stimulus_ | 30 | | | |  |
| Observations | 1258 | | | |  |
| Marginal R^2^ / Conditional R^2^ | 0.000 / 0.151 | | | |  |

*Supplementary Table 16. Norming of selected stimuli for the experiment proper (descriptive statistics).*

| **Stimulus** | **Correct label registered** | **Best fit out of three** | **Mean rating of relatedness** |
| --- | --- | --- | --- |
| bottle | 100% | 80% | 3.80 (0.51) |
| bracelet | 85% | 55% | 3.25 (1.13) |
| brick | 100% | 40% | 3.50 (0.67) |
| broom | 100% | 83% | 3.83 (0.37) |
| button | 95% | 80% | 3.90 (0.30) |
| candle | 100% | 67% | 3.83 (0.37) |
| chair | 100% | 80% | 3.95 (0.22) |
| chopsticks | 85% | 70% | 3.85 (0.36) |
| cushion^a^ | 30% | 50% | 3.50 (0.81) |
| fork | 100% | 90% | 4.00 (0.00) |
| frame | 100% | 50% | 3.80 (0.40) |
| hairband^b^ | 60% | 45% | 3.70 (0.46) |
| hairpin^c^ | 70% | 90% | 3.85 (0.65) |
| hanger | 100% | 50% | 3.85 (0.36) |
| hat | 100% | 75% | 3.95 (0.22) |
| key | 100% | 70% | 4.00 (0.00) |
| nail | 90% | 45% | 3.50 (0.96) |
| paperclip | 90% | 80% | 3.90 (0.44) |
| pencil | 100% | 70% | 3.85 (0.48) |
| rope | 100% | 70% | 3.95 (0.22) |
| ruler | 100% | 55% | 4.00 (0.00) |
| scissors | 100% | 55% | 3.70 (0.56) |
| screwdriver | 100% | 50% | 3.90 (0.30) |
| shoe | 100% | 50% | 3.71 (0.46) |
| spoon | 100% | 70% | 3.85 (0.36) |
| toothbrush | 100% | 60% | 3.55 (0.81) |
| towel | 100% | 75% | 4.00 (0.00) |
| tyre | 100% | 100% | 4.00 (0.00) |
| umbrella | 100% | 92% | 4.00 (0.00) |
| cork* | 100% | 60% | 3.85 (0.36) |
| shovel* | 70% | 55% | 3.65 (0.73) |

*used as practice session

^a^ low percentage because participants often used the word “pillow”

^b^ low percentage because of differences in spelling (hair band, hairband)

^c^ low percentage because of differences in spelling (hair pin, hairpin)

# Supplementary Information

## Instructions for raters

| **Rating scale** | **Description** |
| --- | --- |
| **n/a** | Unfinished idea / unintelligible |
| **0** | Unoriginal idea |
| **1** | Not very original but a bit uncommon, not directly related to the intended use of the object |
| **2** | Quite original, quite uncommon |
| **3** | Original, uncommon |
| **4** | Very original, very rare (wow!) |
| **10** | The idea is nonsensical / unrealistic / impossible |

Your task is to rate the ideas for common and alternative uses for everyday objects. Use the scale above. Use your best judgement. Rate all the rows (don't leave any cells without a rating). Be consistent, that is, if you see the same use for a given object provided more than once in the list, give it the same rating for all examples. If relevant for the condition, please look at the picture next to the table to see what the idea refers to. The provided pictures were the prompts to which some of the ideas were generated. Assess the originality keeping in mind either the picture prompts or the lack thereof (when only a word appeared as a prompt). Please rate an idea description even if it has some spelling problems as long as you can still understand it (for instance, “to protectfromrain” instead of “to protect from rain”). If you can't make out what the description is supposed to mean, type “n/a”. You are encouraged to use the full scale.

Example:

| **earrings** |  |
| --- | --- |
| **0** | jewellery |
| **1** | decoration of a house |
| **2** | to punch a small hole through paper |
| **3** | to attach it to a bag or coat zipper to pull it more easily |
| **4** | to put on a doll as eyes |
| **10** | to eat it for breakfast |
| **n/a** | to use as manc |
